# The Impacts of Water Conservation Strategies on Water Use: Four Case Studies^1^

**DOI:** 10.1111/j.1752-1688.2011.00534.x

**Published:** 2011-08

**Authors:** Yushiou Tsai, Sara Cohen, Richard M Vogel

**Keywords:** water conservation, water demand management, water resource planning, nonparametric statistics, controlled experiments

## Abstract

We assessed impacts on water use achieved by implementation of controlled experiments relating to four water conservation strategies in four towns within the Ipswich watershed in Massachusetts. The strategies included (1) installation of weather-sensitive irrigation controller switches (WSICS) in residences and municipal athletic fields; (2) installation of rainwater harvesting systems in residences; (3) two outreach programs: (a) free home indoor water use audits and water fixture retrofit kits and (b) rebates for low-water-demand toilets and washing machines; and (4) soil amendments to improve soil moisture retention at a municipal athletic field. The goals of this study are to summarize the effectiveness of the four water conservation strategies and to introduce nonparametric statistical methods for evaluating the effectiveness of these conservation strategies in reducing water use. It was found that (1) the municipal WSICS significantly reduced water use; (2) residences with high irrigation demand were more likely than low water users to experience a substantial demand decrease when equipped with the WSICS; (3) rainwater harvesting provided substantial rainwater use, but these volumes were small relative to total domestic water use and relative to the natural fluctuations in domestic water use; (4) both the audits/retrofit and rebate programs resulted in significant water savings; and (5) a modeling approach showed potential water savings from soil amendments in ball fields.

## Introduction

The Ipswich watershed, situated north of metropolitan Boston, MA, has experienced unnaturally low or no flows during some summer months in recent years owing in part, to increases in public water supplies (Canfield *et al.*, 1999; Zarriello and Ries, 2000). The ongoing streamflow depletion has raised awareness of the importance of water demand management among the water authorities, and as a result, the Massachusetts Department of Conservation and Recreation (MDCR) launched a project, funded by the U.S. Environmental Protection Agency (USEPA), in an attempt to identify and pilot strategies that could help restore instream flows to the Ipswich River. In coordination with four communities in the Ipswich watershed, four water conservation projects were designed to simultaneously meet immediate municipal needs and demonstrate innovative water conservation strategies that could be evaluated with real-world data. The four projects are (1) installation of weather-sensitive irrigation controller switches (WSICS) at residences and at municipal athletic fields, (2) installation of rainwater harvesting systems at residences, (3) town-administered programs to provide (a) home indoor water use audits and fixture retrofit kits and (b) rebates for low-water-demand toilets and washing machines, and (4) soil amendments to improve moisture retention and reduce water demand at a municipal athletic field.

The primary goal of this study is to evaluate the effectiveness of four water conservation pilot strategies on water use. As is inherent to many small-scale pilots, the datasets for these demonstration projects tend to be small, variable, and exhibit nonnormal distributions. A secondary goal of this study is to demonstrate the application of mostly nonparametric statistical methods for their ability to enable sensible inferences to be drawn, in some cases, even from the very small samples.

Vickers (2001) has reviewed approaches relating to water conservation strategies for municipal, industrial, and residential uses. Hilaire *et al.* (2008) have summarized factors impacting the efficiency of water use in the urban landscape: water conservation strategies, landscape design, economic and noneconomic incentives, irrigation/water application and reuse technologies, and people-plants relationship. Most previous research on water conservation strategies involves price incentives. Literature on the price elasticity of water use – impact of water price on water demand – is so well developed that meta-analysis is now possible (e.g., see the meta-analysis of 64 previous studies by Dalhuisen *et al.*, 2003). A review of research relating to nonprice water conservation strategies, in which price incentives are not used, reveals fewer studies. We note three general approaches to nonprice water conservation research: (1) behavioral approaches, (2) retrospective analyses, and (3) controlled experiments. Examples of the first approach are provided by Corral-Verdugo and Frias-Armenta (2006) and others who have evaluated the impact of social norms (an understanding of the attitudes and behavior of others) on water conservation behavior. Similarly, Atwood *et al.* (2007) and others have identified the key behavioral, community, and other socioeconomic factors that impact water conservation, such as gender, environmental attitudes, and neighborhood features. Gilg and Barr (2006) have provided a review of research that summarizes behavioral attitudes toward water conservation. Most previous behavioral research on water conservation consists of controlled experimental designs based on a combination of surveys and multivariate statistical analyses.

A second approach to nonprice water conservation research involves a retrospective analysis of previous water use behavior using available data. For example, Kenney *et al.* (2004) showed the importance of water-use restrictions in reducing water demands during a drought experienced by eight Colorado cities. Most retrospective research on nonprice water conservation strategies has developed multivariate relationships for predicting residential water demand as a function of conservation efforts in addition to numerous other factors or explanatory variables. For example, some of the combinations of explanatory variables considered for predicting water demand, in addition to conservation efforts, include price, household appliances, landscape features, metering, and climate (Bamezai, 1995); price, weather, and demographic characteristics (Kenney *et al.*, 2008); price and public information (Wang *et al.*, 1999; Smith and Wang, 2008); price, weather, household income, municipalities, public information, and education (Michelsen *et al.*, 1999); price, public information, weather, household characteristics, water use restriction, and ration (Renwick and Green, 2000); or price, public information, weather, household characteristics, use restriction, ration, and month (Renwick and Archibald, 1998). For those cited studies, the demand elasticity in response to conservation efforts ranged from 0.03 to −4.51 for indoor strategies and 0 (unresponsive) to −4.81 for outdoor strategies.

On the other hand, the price elasticity of water demand reported in previous research on price approaches to water conservation varies. For example, Espey *et al.* (1997) found that price elasticity ranged from −0.02 to −0.75 for 75% of price elasticity estimates, whereas Brookshire *et al.* (2002) found estimates ranging from −0.11 to −1.59, and although Dalhuisen *et al.* (2003) concluded that price elasticity of water demand is relatively elastic, the authors cautioned that price elasticity varied depending on functional form selection, aggregation level, data characteristics, and estimation issues. In conclusion, these studies indicate that the effectiveness of both nonprice and price approaches varied drastically, thus we are unable to judge from previous research whether nonprice or price approaches are more effective. Moreover, Dalhuisen *et al.* (2003) has concluded that price elasticity in East United States is insignificant; therefore, in the context of our analysis, it is probably safe to view economic incentives to be relatively ineffective in comparison with other incentives considered here.

A third approach to nonprice water conservation research, and the approach used here, involves the use of controlled experiments combined with statistical methods. Here controlled experiments are performed with actual water conservation methods. For example, Karpiscak *et al.* (2001) estimated water savings by monitoring a water conservation demonstration house. The water savings reported by Karpiscak *et al.* (2001), however, may not be an accurate response to a single water conservation strategy because the synergistic effects associated with multiple water conservation practices implemented inside the demonstration house were not considered. Buchberger and Wells (1996) monitored residential water demand at four households over a one-year period and used that information to develop stochastic models of residential water demands. Although their work did not deal directly with water conservation efforts, such research could provide important inputs to future water conservation strategies. Mayer *et al.* (2003, 2004) and Ayres Associates (1996) have employed *t*-tests to assess water savings due to various water conservation strategies in an experimental group relative to a control group.

There are a few examples of the type of research performed here, in which designed experiments are used to evaluate the effectiveness of water conservation technologies and programs using hypothesis tests (Ayres Associates, 1996; Mayer *et al.*, 2003, 2004). Those studies employed traditional parametric statistical methods, and the applicability of the *t*-test used in these studies was not assessed by an investigation of probability distributions of the datasets. The researchers assumed that the data arose from a normal distribution without performing normality checks. Here, we are careful to confirm the suitability of statistical methods before their application to controlled experiments to assess the effectiveness of each of four independent water conservation strategies. We begin by providing an overview of the four conservation strategies considered and reviewing the statistical methods employed.

## Methodologies

### Design of Water Conservation Strategies

Four water conservation strategies designed to reduce water use were implemented in the Ipswich River watershed by MDCR, with funding from the USEPA. Due to the critical contribution of outdoor irrigation to the summertime streamflow deficit (Ipswich River Watershed Action Plan, 2003), these water conservation strategies piloted and evaluated here have a strong emphasis on reducing lawn and athletic field irrigation. The installation of WSICS at residences and municipal athletic fields, the installation of rainwater harvesting systems, and the introduction of moisture-retaining soil amendments at an athletic field are all strategies designed to mitigate water withdrawals for irrigation purposes during the summer months. In addition, the home audit/retrofit and appliance rebate programs aim to mitigate withdrawals for indoor water use, year round. Each case study was designed in cooperation with one or more municipality in the watershed, based on an opportunistic assessment of water conservation needs and programmatic resources.

This section, along with Table 1, summarizes the water savings hypothesis and evaluation design for each of the four demonstration projects. The WSICS are designed to only trigger an irrigation cycle when the soil moisture is low, as estimated from regional weather conditions and local rainfall. By delivering water optimally, such technology should reduce overall irrigation demand by eliminating extraneous cycles triggered by automatic timers that are insensitive to weather conditions. The rainwater harvesting systems store rainwater, providing a direct alternative to the use of public drinking water for nonpotable outdoor uses. We thereby anticipated that the systems would reduce demand on household public water consumption. The moisture-retaining soil amendments were designed to extend the time that moisture remains available to the turf roots within the soil. As a result, we anticipated that the field could tolerate reductions in irrigation volume without compromising turf health. The audit/retrofit program was anticipated to reduce water use in participating households by leading to the direct repair of leaks and the replacement of faucets and water fixtures with more efficient alternatives. The rebate program was anticipated to similarly reduce household water use by encouraging the conversion to water-efficient toilets and washing machines.

**TABLE 1:** Summary of Evaluation Design for the Four Water Conservation Strategies

Conservation Strategies and Evaluation Design	Sample Size	Date of Installation or Start of Implemention	Pre Period	Post Period or Simulation Period	Time Period Excluded From Analysis	Confounding Factors
Residential WSICS – Evaluation 1: Comparison of experimental group's outdoor water use prior to and after WSICS installation; weather controlled for by comparing to control group's outdoor water use during same time periods	Control: 71 Experiment: 9 (2 households in the Pilot Group for Evaluation 2 below were excluded from this analysis, as they joined the study in 2006-2007)	Summer of 2005	2001-2004 quarterly water use data from dedicated outdoor water meters	2006-2007 quarterly water use data from dedicated outdoor water meters	2005 water use data were excluded, because 2005 was a transitional year	Participation was voluntary. Self-selection may bias sample toward (1) high initial water users (expect positive savings) or (2) conservative initial water users (expect negative savings)
Residential WSICS – Evaluation 2: Comparison of experimental group's actual water use prior to WSICS installation to simulated water use with WSICS for same period, using known historic weather records	Experiment: 11	Summer of 2005 (9 participants) Summers of 2006 or 2007 (2 participants)	2003-2004 actual annual aggregated outdoor water use	2003-2004 simulated annual aggregated outdoor water use		
Municipal WSICS – Comparison of actual water use prior to WSICS installation to simulated water use with WSICS for same period, using known historic weather records	5 ball fields	Summer of 2005	2003-2004 actual annual aggregated water use	2003-2004 simulated annual aggregated water use		
Rainwater harvesting – Comparison of domestic (public) summer water use prior to and after installation of rainwater harvesting system; comparison of rainwater use and rainfall capture efficiency between 0.76 and 3.03 m^3^ systems	0.76 m^3^ (200-gallon) tank: 26 3.03 m^3^ (800-gallon) tank: 11 1.38 m^3^ (365-gallon) tank: 1 2.27 m^3^ (600-gallon) tank: 1	Mid-April 2006	2004-2005 domestic water use	2006-2007 domestic water use 2006-2007 rainwater use		Substantial difference in scale between domestic and rainwater use
Audit/retrofit and rebate programs – Comparison of winter water use prior to and after audit/retrofit event or installation of one or more rebate-qualifying appliance; participants grouped into mutually exclusive categories by type of participation. Control group used to control for external causes of pre *v*. post differences	Audit/Retrofit: 99 Rebate-toilet: 87 Rebate-washer: 527 Rebate-toilet and washer: 30 Audit/Retrofit and Rebate (toilet and/or washer): 32 Control group: 5,050	Variable, beginning September 2003 for Audit/Retrofits and July 2003 for Rebates	Quarterly winter water use from February 2001 to audit date or appliance installation date Control group established for each pre *v*. post period	Quarterly winter water use from audit date or appliance installation date to May 2007 Control group established for each pre *v*. post period	Only winter quarters included: (began on or after October 19 and ended on or before April 14)	
Soil amendments – Comparison of simulated water use between control and experimental fields during same period, using known historical weather records and known weather triggers for irrigation on both fields	Control: 1 Experiment: 1		2003-2007 simulated water use data for control field	2003-2007 simulated water use data for experimental field		

A summary of evaluation design for all four water conservation strategies is documented in Table 1. This table includes the sample sizes associated with the control and experimental populations, the time periods associated with the installation and the pre- and postexperiment evaluations, the time periods excluded from the analysis, and a list of the confounding factors.

### Statistical Methods

A wide range of statistical methods are considered due to the different experimental designs and nature of the four water conservation strategies, which were designed in accordance with towns’ specific needs and administrative abilities. Nonparametric statistical methods are often recommended over parametric methods (Helsel and Hirsch, 2002) when sample sizes are limited and/or in cases when a probability distribution cannot be determined for the random variable of concern. Here, we used mostly nonparametric hypothesis tests, because most of the datasets were either too small and/or they violated various assumptions required for parametric hypothesis tests to be meaningful. We assumed, throughout our analyses, that the type I error probability *α* was 5%.

We used nonparametric confidence intervals for the true population median because the probability distributions of the original random variables could not be confirmed for small samples. Such confidence intervals for the true population median, shown in many subsequent figures, are used to assess whether the median estimated from one sample differs from the median estimated from another sample. Helsel and Hirsch (2002) suggested that the nonparametric interval for the median can be estimated using the binomial probability distribution. The probability of an observation being above or below the median is equal so that *p* = 0.5. For a sample size *n*, the cumulative probability *p*(*x*) of x observations exceeding the median is then


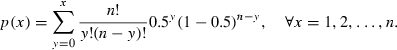
(1)

The lower bound of the interval can be estimated using the (*x* + 1)th smallest observation, where *x* corresponds to *p*(*x*) = 0.025, which reflects a 2.5% probability in each tail of the distribution of *x*. The upper bound of the interval can be estimated using the (*n* − *x*)th smallest observation. The resulting confidence intervals for the median reflect the distributions of the estimates of medians drawn from any dataset of length *n*. For cases where the sample sizes are large (*n* > 20), one may use a normal approximation to the binomial distribution in Equation (1) leading to the rank corresponding to the lower bound of the interval estimate of:


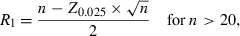
(2)

and the upper bound of the interval estimate is the *R*_*u*_th smallest observation, where


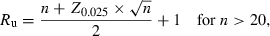
(3)

and *Z*_0.025_ = 1.96.

In some instances, we were able to employ hypothesis tests based on the assumption of a normal distribution. To check whether observations of a sample are normally distributed, the normal probability plot correlation coefficient (PPCC) was computed and checked against its critical value given in table 18.3.3 of Stedinger *et al.* (1993). The normal quantiles were estimated using Blom's unbiased, plotting position for normal variates (Stedinger *et al.*, 1993):


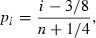
(4)

where *i* is the *i*th observation when ranked in ascending order.

The hypothesis tests used in this study, corresponding to the various types of comparisons, are documented in Table 2. The sign test was chosen over the sign rank test and the paired rank-sum test because the latter two assume a symmetrical distribution of the observations and most of our datasets are asymmetrical.

**TABLE 2:** The Hypothesis Tests Used in This Study Are Presented by Shaded Cells

Comparison Between or Among	One Sample and Two Dependent Samples	Two Independent Samples	More Than Two Independent Samples	More Than Two Dependent Samples
Nonparametric tests	Sign test^1^,^2^	Rank-sum test (or Wilcoxon-Mann-Whitney test)^2^	Kruskal-Wallis test^1^,^2^	Appropriate test is not available^1^
Parametric tests	*t*-test^1^,^2^ Paired *t*-test^1^,^2^	Two-sample *t*-test^1^,^2^	One-way ANOVA^1^,^2^	Two-way ANOVA or multi-way ANOVA^1^

1 Zar (1999)

2 Helsel and Hirsch (2002).

## Water Savings Associated With Water Conservation Strategies

The following sections summarize the effectiveness at reducing water demand of the four water conservation strategies.

### Weather-Sensitive Irrigation Controller Switches

A total of 11 WSICS were evaluated on residential properties and 5 in municipal athletic fields. These devices (Weather Reach WR-7® by Irrisoft®, Logan, UT, USA) contain an on-site rain gage and receive continuous solar radiation, temperature, relative humidity, and wind data from a regional weather station (town of Ipswich) via wireless transmission. Based on this information, the WSICS device is designed to deliver water only when needed by the landscape.

### Residential WSICS

Approximately 150 residences in the town of Reading, MA, have exclusive outdoor water meters. Among this group, nine households that met our experimental group criteria had WSICS installed during the summer of 2005, and two during the following two summers. Criteria included continuous ownership and use of an automatic irrigation system since 2001. An additional 71 households with dedicated outdoor meters meeting these criteria were selected as the control group. For this analysis, quarterly outdoor water use records were obtained from the Reading Water Department for all households in the study from January 2001 through November 2007.

**FIGURE 1:**
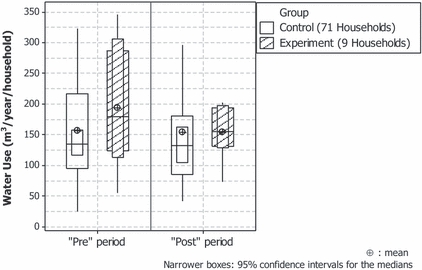
BoxPlots Comparing Annual Outdoor Water Use in the Control and Experimental Groups in Both the “Pre” (2001 to 2004) and “Post” (2006 to 2007) Periods.

For the nine residences whose WSICS was installed in 2005, a single value representing historic (“pre”) water use (pre-experimental condition) was obtained by averaging the annual outdoor water use from 2001 to 2004, and a single value representing water use during the experimental period (“post”) was obtained by averaging the annual outdoor use from 2006 to 2007. Data from 2005 were excluded from the analysis due to this being a transitional year. Because a PPCC normality test determined that the control group was not well approximated by a normal distribution, the nonparametric rank-sum hypothesis test was used to compare the water use of both the control and experimental groups as shown in Figure 1. There is no statistically significant difference between the water use of the control and experimental groups in either the “pre” or “post” periods, which can be seen visually in Figure 1 with the overlapping confidence intervals. However, a visual assessment of Figure 1 also suggests that the WSICS may have reduced the variability of water use among the experimental group, especially among high water users.

**FIGURE 2:**
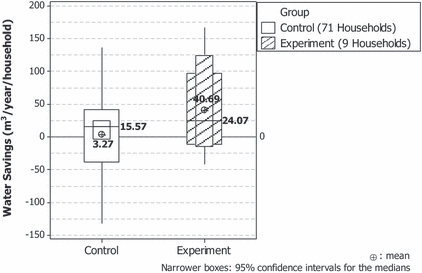
BoxPlots Showing Water Savings During the “Post” Period Relative to the “Pre” Period, in Both Groups. For each household, this value represents “post” period water use subtracted from “pre” period water use. A value <0 implies more water was used during the “post” than “pre” period.

**FIGURE 3:**
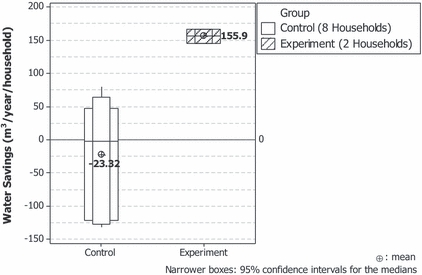
Comparison of the Annual Outdoor Water Savings Between the Control and Experimental Groups Among the 90th Percentile of “Pre” Period (2001 to 2004) Water Users (annual use >261.6 m^3^).

Rank-sum tests applied to the rainfall records from a nearby water treatment plant suggest that typical total rainfall and number of days of rain between May 15 and October 15 (the approximate irrigation season) were statistically indistinguishable during the “pre” and “post” periods. Thus, we were comfortable calculating “savings” for each household by subtracting the “post” from the “pre” period water use. The results of a rank-sum test do not show that the water savings for households with the WSICS were different than for the control group. The large range associated with the confidence interval (Figure 2) for the median of the experimental group is due to the small experimental sample size and large variation in response to the WSICS installation within the group. Nevertheless, Figure 2 illustrates that although the average household in the control group saw a drop in water demand of 3.27 m^3^/year between the two time periods, the average WSICS household saw a reduction of 40.69 m^3^/year. Although this difference is not statistically significant, it reflects the fact that households with high “pre” period water demand saw a large reduction in water use postinstallation. As shown in Figure 3, when only the highest “pre” period water users (90th percentile; annual use >261.6 m^3^) are included in the analysis, the water savings for the experimental group is significantly greater than the control group. These results suggest that households with high irrigation water demands are more likely to reduce their water use due to the WSICS installations. Our analysis also highlights the importance of increasing the sample size of the experimental group of households in any future studies.

### Retrospective Analysis

A retrospective analysis of the WSICS compared actual outdoor water used by each experimental household in 2003 and 2004 to the estimated volume of water that would have been applied by the WSICS during that same period. This analysis required calculating the number of irrigation cycles that would have been triggered for each system, based on: (1) weather data from that period, (2) the algorithm used by the WSICS units to trigger irrigation cycles based on weather data, and (3) each system's individual “evapotranspiration (ET) threshold.” ET thresholds are used to set the tolerance for how much estimated ET should be allowed before an irrigation cycle is triggered to replenish the loss. The number of triggered irrigation cycles was then converted to a volume for each household by multiplying it by the appropriate per-cycle volume. The latter was determined at each residence by reading the water meter before and after a test irrigation cycle. This approach was only applied to 2003 and 2004 to coincide with the years for which the extensive weather data needed in the algorithm was available. A PPCC normality hypothesis test suggests that the nonparametric sign test is preferred over a parametric test for assessing the difference between the actual and simulated water uses. Although positive overall mean and median water savings (22.60 and 29.28 m^3^/household/year, respectively) are reported when comparing simulated with actual use, we conclude from the nonparametric sign test that the savings is not significantly different from zero, owing to the large variation in the small sample. When this analysis is applied only to water users with high actual water use (use >261.6 m^3^), during the years their use exceeded this threshold, the average savings is statistically significant at 135.8 m^3^/household/year. However, this sample consisted only of one year of data for each of three households.

In summary, two approaches were used: (1) comparing outdoor water use in households where WSICS were installed to outdoor water use in control households, both prior to and after installation; and (2) the retrospective analysis, comparing actual water use to theoretical water use had the WSICS been installed in 2003 and 2004. Both approaches confirm that even though overall water savings for the experimental group is greater than that for the control group, the difference in the savings between the two groups was not statistically significant owing to the highly variable savings in the experimental group. WSICS were, however, likely to result in water savings when installed at residences with high outdoor water demands. Although we did not assess the efficiency of individual watering regimes prior to WSICS installation, the significant response to the systems among the highest water users suggests over-watering by these households prior to the WSICS installation, as WSICS systems are designed specifically to reduce unnecessary irrigation.

### Municipal WSICS

**FIGURE 4:**
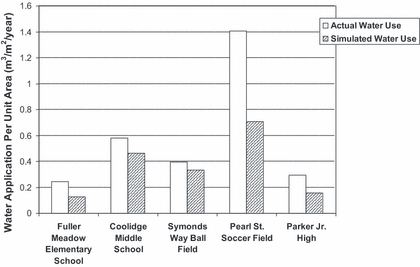
Actual Water Use (without WSICS) and Simulated Water Use (with WSICS) Aggregated for 2003 and 2004 for Each Ball Field.

**FIGURE 5:**
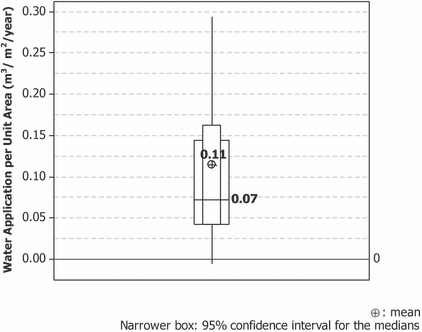
Box Plot of Theoretical Water Savings (actual–simulated water use) for Each Ball Field, Each Year (2003 and 2004). Mean per-unit-area savings is 0.11 m^3^/m^2^ per year (equal to 121,000 gallons/acre/year).

In addition to residential WSICS, five municipal athletic fields across two municipalities (Reading and Middleton, MA) were equipped with WSICS in the summer of 2005. A retrospective analysis was conducted using the same methodology as described above for the residential participants. Hypothetical water use was derived by simulating irrigation triggers that would have been signaled by the WSICS, had they been installed during 2003 and 2004, using weather records from that period and each field's WSICS ET thresholds and irrigation cycle volumes. This simulated use was compared with actual water use for each of the five fields aggregated for 2003 and 2004 (Figure 4). Theoretical water savings were obtained by subtracting simulated use from actual use for each field for each year. Nonparametric tests were used again due to a sample size of 10 (two years each, for five fields). The sign test indicates that a significant positive water savings would have resulted from the WSICS installations. A box plot of the theoretical water savings (Figure 5) indicates that this statistically significant average savings was approximately 0.11 m^3^/m^2^/year (equal to 121,000 gallons/acre/year).

### Rainwater Harvesting

**FIGURE 6:**
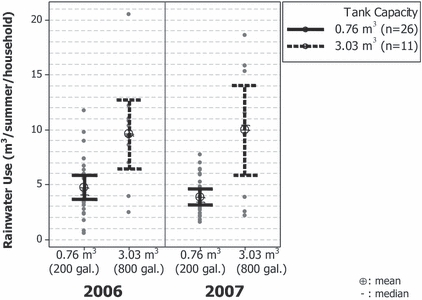
The Data and 95% Confidence Intervals for the Mean of the Total Rainwater Used From Both Sizes of Harvesting Systems During the Summer Watering Seasons of 2006 and 2007.

Rainwater harvesting systems are designed to capture runoff from rooftops and store the water for nonpotable uses, such as lawn and garden watering. One intent of such systems is to reduce demand on public water supplies by replacing potable water that would otherwise be used for these outdoor purposes. A total of 39 rainwater harvesting systems were installed on residential properties mid-April 2006 in the town of Wilmington, MA, based on a lottery among 150 interested households. The systems consist of a storage tank, a pressure pump to aid in water distribution, a spigot for a hose, and a water meter to measure flow pumped from the tanks. Two different sizes of storage tanks were installed: twenty-eight 0.76 m^3^ (200-gallon) and eleven 3.03 m^3^ (800-gallon) tanks. Two of the participants with 200-gallon tanks upgraded their storage capacity to 1.38 m^3^ (365 gallons) and 2.27 m^3^ (600 gallons), respectively, using their own funds. Except where otherwise noted, the households with upgraded systems were excluded from the analyses. The rainwater systems were in use during the summers of 2006 and 2007. Total rainwater use from the time each system was turned on in the spring to when it was decommissioned in the fall was recorded for each household for 2006 and 2007. The distribution of the rainwater use observations for both groups is well-approximated by a normal distribution. All households used the rainwater systems, and a two-sample Student's *t* hypothesis test on sample means indicates that those with 3.03 m^3^ tanks used significantly more rainwater than those with 0.76 m^3^ tanks (Figure 6).

**FIGURE 7:**
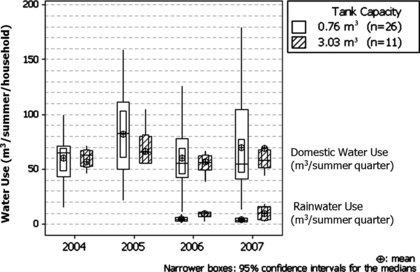
Comparison of Scale Between Household Domestic Water Use and Rainwater Use.

To assess whether the use of rainwater resulted in a decrease in domestic water use, domestic water use before and after the installation of the rainwater harvesting system was compared for each residential participant. The visual comparison of the domestic water use and the rainwater use in Figure 7 shows that the volumes of rainwater used were generally less than the fluctuation in domestic water use from year to year, making reductions in domestic water use due to rainwater difficult to discern. A rank-sum test confirmed that, regardless of the size of the tanks, rainwater systems could not be shown to impact summer domestic water use.

However, a written survey completed by all participants who attended a meeting at the conclusion of the study suggests qualitatively that rainwater was a frequent substitute for domestic water among the rainwater harvesting participants. The survey asked participants to allocate the proportion of the rainwater they used across seven usage activities (one category was defined flexibly as “other” to capture uncommon uses) and to state for each whether they would have used an equivalent or greater amount of domestic water for that purpose if they did not have access to stored rainwater. All respondents (19 of 37 households that were in the program; i.e., 50% of participants) estimated that at least some of their rainwater uses were direct substitutes for domestic water that they otherwise would have used for the same purpose.

**FIGURE 8:**
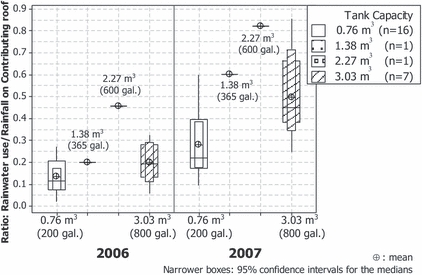
Rainfall Capture Efficiency in, 2006 and 2007. When the sample size n = 1, the interquartile box and confidence interval for the median cannot be determined.

Twenty-five households were able to provide estimates of the roof area contributing to their rainwater collection system. For each of these households, the total volume of rain falling on the contributing area was estimated by multiplying contributing area by daily rainfall depth recorded at a nearby facility for the days the system was in use. Rainfall capture efficiency was defined as the ratio of total volume of rainwater used relative to the total volume of rain that fell on the contributing roof area. Each household has a unique rainfall capture efficiency, based on the combined influences of system storage capacity, frequency of system use, and the pattern (distribution, intensity, etc.) of rainfall events. A rank-sum test of “rainfall capture efficiency” by system size (Figure 8) suggests that, in 2007, households with 800-gallon systems had statistically higher efficiencies than those with 200-gallon systems, whereas in 2006 the two groups had statistically equivalent efficiencies. The efficiencies of both groups improved in 2007 relative to 2006, which might be explained by a difference in rainfall patterns between the two years or might indicate a learning curve as participants get used to system operation. As a final observation, the two households with modified systems (365- and 600-gallon systems) demonstrated a relatively high rainfall capture efficiency among all the study participants. A possible explanation is that the participants who took extra care to tailor their systems to their specific needs were able to increase their systems’ efficiency.

### Residential Audit/Retrofit and Water Conservation Appliances Rebates

As part of a town-wide water conservation plan, in September of 2003, the town of Reading, MA, began offering water customers free indoor water use audits and water saving retrofit devices tailor-made to the results of the audits. The town also began offering customers rebates for eligible water-efficient appliances (washing machines and toilets) purchased on or after July 1, 2003. The purpose of this study was to evaluate the effectiveness of these two programs at reducing town-wide water demand and the water demand of those households who chose to participate in either or both programs. Only winter water use data were evaluated to isolate indoor water use and eliminate the confounding effect of year-to-year weather variability on water use during the irrigation season.

Participating households were grouped into five mutually exclusive categories of participation: (1) audit/retrofit (AR), (2) audit/retrofit and any type of rebate(s) (AR&R), (3) rebate-toilet(s) (RT), (4) rebate-washing machines(s) (RW), and (5) rebate-toilet(s) and washing machine(s) (RT&W). Participants in the same category should not be interpreted to have exactly the same level of participation. For example, the numbers of low-flush toilets for any two households in the group RT may be different, and the number of retrofit devices installed among households in the group AR is variable. This variability did not hinder analysis, as the intent of the study was not to evaluate savings associated with individual technologies, but rather savings resulting from the programs as a whole, which naturally include varying levels of participation.

Quarterly water use records for the entire town were obtained from February 2001 through May 2007. To isolate indoor water use, only quarters that began on or after October 19 and ended on or before April 14 of any year were included in the analysis. For each household, records dated before the installation of a qualifying rebate device or date of audit are regarded as “pre” winter use, whereas those recorded after are “post” winter use. Savings was determined by subtracting the average of the “post” use records from the average of the “pre” use records. To control for factors other than participation in the water conservation program that might trigger a change in water use patterns, households that did not participate in any program were included in a control group. However, as participating households initiated their participation across different years during the study window, a single date could not be selected to separate “pre” and “post” time periods for the control group. Therefore, we analyzed the control group four times to coincide with the variable points of initiation for the participating households. Specifically, “pre” minus “post” water use was calculated for the control group using each of the following four pre *v*. post groupings of years: (1) 2001-2002 *v*. 2003-2007, (2) 2001-2003 *v*. 2004-2007, (3) 2001-2004 *v*. 2005-2007, and (4) 2001-2005 *v*. 2006-2007.

**FIGURE 9:**
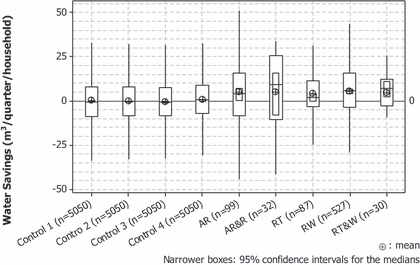
Winter Water Savings Among the Five Different Water Conservation Treatment Categories and the Control Group, Analyzed Four Ways. Values <0 imply an increase in water use after installing a water conservation device or receipt of an audit and retrofit kit. The five treatment categories are: audit/retrofit (AR); audit/retrofit and any type of rebate(s) (AR&R); rebate-toilet(s) (RT); rebate-washing machines(s) (RW); and rebate-toilet(s) and washing machine(s) (RT&W).

The normal PPCC hypothesis test results suggested that nonparametric hypothesis tests are preferred. Sign tests showed statistically significant winter water savings in each conservation program category except AR&R (Figure 9 and Table 3a). However, the AR&R households (those participating in both the audit/retrofit and rebate programs) did demonstrate the highest median and second-highest average savings among the categories. The small sample size of this group likely explains our inability to detect a statistically significant savings for this category. In contrast to the households participating in the conservation programs, the control group households showed no statistically significant changes in water use for any of the time frames defined.

**TABLE 3:** (a) Sample Size, Mean and Median Water Savings for Each of the Five Participation Categories, (b) Participation Rates and Town-Wide Savings for Audit/Retrofit and Appliance Rebate Programs

	Savings (m^3^/quarter/household)				
	
(a)	AR	AR&R	RT	RW	RT&W
*N*	99	32	87	527	30
Mean water savings	4.93	5.01	3.94	5.38	4.58
Median winter water savings	3.96	9.20	1.89	5.66	7.08

			**Water Savings (m^3^/quarter/households)**		
				
**(b)**	**N**	**Participation Rate Based on Number of Households in Town (8,436)**	**Mean**	**Median**	**Town-Wide Savings (m^3^/quarter)**
All levels of participation	775	0.092	5.11	5.10	3,950
AR	99	0.012	4.93	3.96	
AR&R	32	0.004	5.01	9.20	
Combined RT, RW, RT&W	644	0.076	5.15	5.19	

To evaluate the effect of the two outreach programs on town-wide water use, the overall per-household median savings for participating at any level in either program was multiplied by the number of participating households (Table 3b). The town saved 3,950 m^3^/quarter as a result of implementing both programs. Town-wide participation rates are shown for each program and for those participating in both programs (number of participating households/number of households in town). Participation rates are an important factor in estimating the overall savings that another town might be able to achieve by implementing similar programs. However, it should be noted that Reading saw waves of new participation each time the town conducted concerted outreach efforts during the course of the programs. We can assume, then, that the participation rates observed in Reading are closely related to the particular level of outreach effort exerted by the town, and it follows that other towns might be able to increase participation rates with more intensive outreach efforts.

### Soil Amendments in Ball Fields

A portion of an 8-acre municipal athletic field complex in the town of North Reading, MA, was redeveloped to maximize infiltration and minimize irrigation requirements and application of fertilizer and pesticides by employing the following techniques: (1) soil enhancement with zeolite, an additive that retains moisture and nutrients; (2) use of drought-resistant turf; and (3) installation of a WSICS (see section on Weather-Sensitive Irrigation Controller Switches). The adjacent field, which has identical solar orientation, drainage patterns, and original soil profile, received only the latter two treatments and was used as a control to evaluate the effectiveness of the zeolite additive.

The field manager progressively adjusted the WSICS ET thresholds for each field in order to identify the most conservative watering scheme that could still maintain healthy turf. These thresholds set the tolerance for how much estimated ET is allowed before an irrigation cycle is triggered. The optimal thresholds of the zeolite and control fields were found to be 0.89 cm (0.35 inches) and 0.64 cm (0.25 inches), respectively. These settings were used to simulate the number of irrigation cycles that the WSICS would have applied to each field over the five-year period from 2003 to 2007, using historic weather data (see Retrospective Analysis under Weather-Sensitive Irrigation Controller Switches for methodology). The number of cycles was then converted to a total annual volume, based on the respective per-cycle volumes measured for each field. Savings was defined by subtracting the total per-acre irrigation volume applied to the zeolite field from that applied to the control field, for each year. The optimum settings resulted in an estimated average annual per-unit-area savings of approximately 3.59 cm^3^/cm^2^ (38,000 gallons/acre), or 37% (Table 4). Such substantial savings suggest that zeolite soil amendments may prove to be a very effective means to reduce irrigation demands of athletic fields. However, these results are highly dependent on the optimal ET thresholds observed for each field, based on trial and error and field observation over the course of a few months. To further refine the expected savings achievable through zeolite soil amendments, optimal watering thresholds could be verified by the use of soil moisture sensors. Additionally, observations over a longer time period that encompass greater variability of weather patterns would help verify optimal ET thresholds and refine long-term savings estimates.

**TABLE 4:** Simulated Irrigation Volumes Applied to Zeolite and Control Fields (2003 to 2007)

		Simulated Volumes in Year (cm^3^/cm^2^/year)					
			
	Threshold (cm)	2003	2004	2005	2006	2007	Mean
Zeolite field	0.89	3.68	5.11	8.37	5.35	9.30	6.36
Control field	0.64	9.08	7.92	11.62	7.90	13.25	9.95
Savings (control-zeolite)		5.40	2.80	3.25	2.56	3.95	3.59
% Savings (savings/control)		59.52	35.40	28.00	32.35	29.82	37.02

## Conclusions

The overall goal of this study was to evaluate the effectiveness of four water conservation pilot strategies on water use. As is inherent to many small-scale pilots, the datasets for these demonstration projects tend to be small, variable, and exhibit nonnormal distributions. A secondary goal of this study was to demonstrate the application of mostly nonparametric statistical methods for their ability to enable sensible inferences to be drawn, in some cases, even from the very small samples.

Statistical hypothesis tests combined with controlled water conservation experiments were used to evaluate water savings associated with four water conservation strategies implemented in communities in the Ipswich watershed in Massachusetts, designed for their combined ability to meet an immediate municipal need and pilot an innovative conservation strategy. Our review of the literature revealed that controlled water conservation experiments combined with nonparametric statistical analyses of the type performed here are not commonly reported. Instead, most previous research has focused on retrospective statistical analyses of water use as well as studies that sought to elucidate behavior and attitudes concerning various water conservation strategies. Our overall findings for each of the four water conservation programs are as follows:

1. *Weather-sensitive irrigation controller switches*: Residential water use patterns were variably impacted by the addition of the WSICS, with some participants showing a decrease and others showing an increase in water use. The WSICS appeared to reduce the variability of water use among residential participants, most notably by causing a reduction in water use of the highest historical water users. Our findings underscore that initial water use patterns are likely to be a prominent factor in determining whether water use will increase or decrease after WSICS installation in a residential setting. Water users who rely on inefficient watering regimes, historically, are more likely to benefit from the WSICS, which may explain why the participants in our study with the highest historical water use showed large and statistically significant water savings after installing the WSICS. In contrast to the residential setting, WSICS installations at municipal athletic fields resulted in consistent reductions in water application, with an average savings of 0.11 m^3^/m^2^/year (121,000 gallons/acre/year). This suggests that, prior to installation of WSICS, ball fields in our study were more consistently overwatered than residential lawns. This is not surprising, given that towns generally require a high level of turf performance on their athletic fields but lack the staff to frequently adjust irrigation settings in response to weather (such as reducing irrigation volumes after or in anticipation of rain events). To ensure sufficient irrigation without frequent adjustments, systems are set to water frequently, regardless of need. Strict standards for turf performance and limited staff resources are common in municipal settings, suggesting that the savings observed at ball fields in this study are likely transferable to other ball field sites.
2. *Rainwater harvesting*: Rainwater was used for outdoor purposes by all participants, and those with 3.03 m^3^ systems (800 gallons) used significantly more than those with 0.76 m^3^ systems (200 gallons). Annual volumes of rainwater used were small compared with domestic water use, and reductions in domestic water use as a result of substitution with rainwater could not be discerned amidst the background fluctuations in domestic water use from year to year. However, a participant survey suggested that for every household, at least some of the rainwater used was a direct substitute for domestic water that would have been used for the same purpose. Rainfall capture efficiency was measured as the ratio of rainwater used relative to the rain that fell on the contributing roof area during the months of system operation. Efficiency of both size systems improved in the program's second year, which may indicate different rainfall patterns between the two years or that there is a learning curve as participants got used to system operation. In the second year, the larger systems were more efficient than the smaller systems, whereas they were statistically equivalent the first year. A possible explanation is that as rainfall capture efficiency improves, the impact of system size becomes more pronounced. Two households that modified their systems’ size were among the most efficient, suggesting that efficiency may be improved by tailoring one's system to one's needs.
3. *Residential audit/retrofit and water conservation appliance rebates*: Participation in two town-administered water conservation programs (a. free indoor water use audits and fixture retrofit kits; b. low flow toilet and washing machine rebates) was divided into five categories. Four resulted in modest but significant positive water savings averaging between 3.94 and 5.38 m^3^/quarter/household. Although the fifth participation category (participation in both programs) showed no statistically significant water savings, this group's median and mean savings were ranked the highest and second-highest, respectively, among all five categories. The finding of nonstatistically significant savings of this group appeared to result from the small sample size and large variation in water savings among the participants. In the first four years of program implementation, 9.2% of the town's households participated in one or both of the programs, resulting in an overall average savings of approximately 3,950 m^3^/quarter for the town.
4. *Soil amendments in ball field*: The addition of a moisture and nutrient-retaining additive, zeolite, to the soil of a ball field resulted in healthy turf with less water applied than to an adjacent control field. Based on observed irrigation requirements, the zeolite material was estimated to save approximately 3.59 cm^3^/cm^2^/year (38,000 gallons/acre/year). This represents a reduction of 37% in irrigation volume, suggesting promising water savings from zeolite soil amendments.

Future research on all of the above strategies could be used to verify or refine the results reported here. To address the specific constraints encountered in this study, the following approaches are suggested. WSICS should be evaluated with larger residential sample sizes and include an assessment of historic irrigation efficiency. Additional size categories of rainwater harvesting systems should be evaluated for rainfall capture efficiency under a variety of rainfall conditions and further investigation should be made into the ability of such systems to reduce domestic water use. Town-administered water conservation programs such as Reading's should continue to be evaluated over longer time frames to better understand the long-term potential for savings among participating households and at the town level. Lastly, turf health on the soil-amended and control ball fields was determined by visual inspection. Future research should employ a more sophisticated method for comparing the turf health.

## References

[b1] Atwood C, Kreutzwiser R, de Loe R (2007). Residents’ Assessment of an Urban Outdoor Water Conservation Program in Guelph, Ontario. Journal of the American Water Resources Association.

[b2] Ayres Associates (1996). An Evaluation of Davis Islands Landscape Irrigation System Conservation Program.

[b3] Bamezai A (1995). Application of Difference-in-Difference Techniques to the Evaluation of Drought-Tainted Water Conservation Programs. Evaluation Review.

[b4] Brookshire DS, Burness HS, Chermak JM, Krause K (2002). Western Urban Water Demand. Natural Resources Journal.

[b5] Buchberger SG, Wells GJ (1996). Intensity, Duration and Frequency of Residential Water Demands. Journal of Water Resources Planning and Management – ASCE.

[b6] Canfield S, Claessens L, Hopkinson C, Rastetter E, Vallino J (1999). Long-Term Effect of Municipal Water Use on the Water Budget of the Ipswich River Basin. Biological Bulletin.

[b7] Corral-Verdugo V, Frias-Armenta M (2006). Personal Normative Beliefs, Antisocial Behavior and Residential Water Conservation. Environment and Behavior.

[b8] Dalhuisen JM, Florzzx RJGM, de Groot HLF, Nijkamp P (2003). Price and Income Elasticities of Residential Water Demand: A Meta-Analysis. Land Economics.

[b9] Espey M, Espey J, Shaw WD (1997). Price Elasticity of Residential Demand for Water: A Meta-Analysis. Water Resources Research.

[b10] Gilg A, Barr S (2006). Behavioural Attitudes Towards Water Saving? Evidence From a Study of Environmental Actions. Ecological Economics.

[b11] Helsel DR, Hirsch RM (2002). *In*: Statistical Methods in Water Resources. Techniques of Water-Resources Investigations Book 4, Hydrologic Analysis and Interpretation.

[b12] Hilaire RS, Arnold MA, Wilkerson DC, Devitt DA, Hurd BH, Lesikar BJ, Lohr VI, Martin CA, McDonald GV, Morris RL, Pittenger DR, Shaw DA, Zoldoske DF (2008). Efficient Water Use in Residential Urban Landscapes. Hortscience.

[b13] Ipswich River Watershed Action Plan (2003). A Publication of the Massachusetts Executive Office of Energy and Environmental Affairs. http://www.mass.gov/Eoeea/docs/eea/water/wap_ipswich_2003.pdf.

[b14] Karpiscak MM, France GW, DeCook KJ, Brittain RG, Foster KE, Hopf SB (2001). Casa Del Agua: Water Conservation Demonstration House 1986 Through 1998. Journal of the American Water Resources Association.

[b15] Kenney DS, Goemans C, Klein R, Lowrey J, Reidy K (2008). Residential Water Demand Management: Lessons From Aurora, Colorado. Journal of the American Water Resources Association.

[b16] Kenney DS, Klein RA, Clark MP (2004). Use and Effectiveness of Municipal Water Restrictions During Drought in Colorado. Journal of the American Water Resources Association.

[b17] Mayer PW, DeOreo WB, Towler E, Lewis DM (2003). Residential Indoor Water Conservation Study: Evaluation of High Efficiency Indoor Plumbing Fixture Retrofits in Single-Family Homes in the East Bay Municipal Utility District Service Area.

[b18] Mayer PW, DeOreo WB, Towler E, Martien L (2004). Tampa Water Department Residential Water Conservation Study: The Impacts of High Efficiency Plumbing Fixture Retrofits in Single-Family Homes.

[b19] Michelsen AM, McGuckin JT, Stumpf D (1999). Nonprice Water Conservation Programs as a Demand Management Tool. Journal of the American Water Resources Association.

[b20] Renwick ME, Archibald SO (1998). Demand Side Management Policies for Residential Water Use: Who Bears the Conservation Burden?. Land Economics.

[b21] Renwick ME, Green RD (2000). Do Residential Water Demand Side Management Policies Measure Up? An Analysis of Eight California Water Agencies. Journal of Environmental Economics and Management.

[b22] Smith WJ, Wang YD (2008). Conservation Rates: The Best ‘New’ Source of Urban Water During Drought. Water and Environment Journal.

[b23] Stedinger JR, Vogel RM, Foufoula-Georgiou E, Maidment R (1993). Frequency Analysis of Extreme Events. *In*: Handbook of Hydrology.

[b24] Vickers A (2001). Handbook of Water Use and Conservation: Homes, Landscapes, Business, Industries, Farm.

[b25] Wang YD, Song JS, Byrne J, Yun SJ (1999). Evaluating the Persistence of Residential Water Conservation: A 1992-1997 Panel Study of a Water Utility Program in Delaware. Journal of the American Water Resources Association.

[b26] Zar JH (1999). Biostatistical Analysis.

[b27] Zarriello PJ, Ries KG (2000). A Precipitation-Runoff Model for the Analysis of the Effects of Water Withdrawals on Streamflow.

